# Reduced incidence of interstitial pneumonitis after allogeneic hematopoietic stem cell transplantation using a modified technique of total body irradiation

**DOI:** 10.1038/srep36730

**Published:** 2016-11-10

**Authors:** Yun Chiang, Cheng-Hong Tsai, Sung-Hsin Kuo, Chieh-Yu Liu, Ming Yao, Chi-Cheng Li, Shang-Yi Huang, Bor-Sheng Ko, Chien-Ting Lin, Hsin-An Hou, Wen-Chien Chou, Jia-Hau Liu, Chien-Chin Lin, Shang-Ju Wu, Szu-Chun Hsu, Yao-Chang Chen, Kai-Hsin Lin, Dong-Tsamn Lin, Hsien-Tang Chou, Meng-Yu Lu, Yung-Li Yang, Hsiu-Hao Chang, Ming-Chih Liu, Xiu-Wen Liao, Jian-Kuen Wu, Sheng-Chieh Chou, Chieh-Lung Cheng, Chien-Yuan Chen, Woei Tsay, Hwei-Fang Tien, Jih-Luh Tang, Yu-Hsuan Chen

**Affiliations:** 1 Division of Radiation Oncology, Department of Oncology, National Taiwan University Hospital, Taipei, Taiwan; 2 Division of Hematology, Department of Internal Medicine, National Taiwan University Hospital, Taipei, Taiwan; 3 Tai-Cheng Stem Cell Therapy Center, National Taiwan University, Taipei, Taiwan; 4 Genome and Systems Biology Degree Program, National Taiwan University, Taipei, Taiwan; 5 Biostatistics Consulting Laboratory, School of Nursing, National Taipei University of Nursing and Health Sciences, Taipei, Taiwan; 6 Depart of Laboratory Medicine, National Taiwan University Hospital, Taipei, Taiwan; 7 Graduate Institute of Clinical Medicine, College of Medicine, National Taiwan University, Taipei, Taiwan; 8 Division of Hematology/Oncology, Department of Pediatrics, National Taiwan University Hospital, Taipei, Taiwan; 9 Department of Pathology, National Taiwan University Hospital, Taipei, Taiwan; 10 Department of Medical Imaging and Radiological Technology, Yuanpei University of Medical Technology, Hsinchu, Taiwan

## Abstract

Allogeneic hematopoietic stem cell transplantation is a curative-intent treatment for patients with high-risk hematologic diseases. However, interstitial pneumonitis (IP) and other toxicities remain major concerns after total body irradiation (TBI). We have proposed using linear accelerators with rice-bag compensators for intensity modulation (IM-TBI), as an alternative to the traditional cobalt-60 teletherapy with lung-shielding technique (Co-TBI). Patients who received a TBI-based myeloablative conditioning regimen between 1995 and 2014 were recruited consecutively. Before March 2007, TBI was delivered using Co-TBI (n = 181); afterward, TBI was administered using IM-TBI (n = 126). Forty-four patients developed IP; of these cases, 19 were idiopathic. The IP-related mortality rate was 50% in the total IP cohort and 63% in the idiopathic subgroup. The 1-year cumulative incidences of IP and idiopathic IP were 16.5% and 7.4%, respectively; both rates were significantly higher in the Co-TBI group than in the IM-TBI group. Multivariate analysis revealed that Co-TBI was an independent prognostic factor for both total and idiopathic IP. In the acute myeloid leukemia subgroup, patients with different TBI techniques had similar outcomes for both overall and relapse-free survival. In conclusion, IM-TBI is an easy and effective TBI technique that could substantially reduce the complication rate of IP without compromising treatment efficacy.

Total body irradiation (TBI) has an established role in the conditioning regimens that are administered before allogeneic hematopoietic stem cell transplantation (HSCT)^1^,^2^,^3^,^4^. There are several rationales for administering TBI before HSCT: to lower the risk of engraftment failure through TBI’s immunosuppressive effects on the host; to eradicate residual malignant cells through its cytotoxic effects, especially in sanctuary sites like the central nervous system (CNS) and testes, which are difficult for chemotherapy to access; and to eradicate cells with genetic disorders, such as in cases of Fanconi’s anemia or Wiskott-Aldritch syndrome. Previous studies of conditioning regimens for acute leukemia have found that, as compared with chemotherapy alone, the combination of TBI and chemotherapy provided non-inferior outcomes^5^,^6^,^7^,^8^,^9^,^10^,^11^,^12^,^13^ and a lower CNS relapse rate^9^, even without cranial boost^14^. However, the addition of TBI would be expected to increase the risk of interstitial pneumonitis (IP), which has been the major dose-limiting toxicity. The incidence of IP has variously been documented as 10 to 85%^9^,^15^,^16^,^17^ and, strikingly, approximately half of these IP cases were fatal^16^,^17^. Many risk factors for IP were previously identified, including single-fraction TBI^18^, TBI with a higher total dose^18^, TBI with a higher dose rate^15^,^19^, and acute graft-versus-host disease (GvHD)^15^,^16^,^17^,^18^,^20^,^21^,^22^,^23^. To date, there have been relatively few studies of attempts to lower the IP rate by improving radiation techniques.

During the era of cobalt-60 teletherapy (Co-TBI) at our institute (1995 to March 2007), TBI was delivered anteroposteriorly using parallel opposed fields with lung blocks to lower the lung dose^24^. Since 2007, linear accelerators have become predominant, and we have developed a bilateral TBI technique that uses rice-bag compensators as intensity modulators (IM-TBI). Both techniques were delivered with a hyperfractionation schedule and a fixed dose of 12 Gray (Gy). The aim of this study is to evaluate the efficacy and complication rate of the newly developed TBI method.

## Results

### Patient characteristics

Among the 307 patients enrolled in this study, 181 patients received Co-TBI and 126 patients received IM-TBI. Data on forced vital capacity (FVC) and forced expiratory volume in 1 s (FEV1) were available for all patients. Information on total lung capacity (TLC) and carbon monoxide diffusing capacity of the lungs (DLCO) were available for 228 and 195 patients, respectively. The Co-TBI and IM-TBI groups were comparable in terms of age, sex, and the proportion of cases with complete remission (CR) at the time of HSCT (P = 0.375, 0.907, and 0.811, respectively). As compared with the Co-TBI group, more of the patients in the IM-TBI group had acute leukemia (86% vs. 75%, P = 0.022), initial CNS involvement (30% vs. 5%, P < 0.001), and GvHD of either the acute or chronic type (66% vs. 35%, P < 0.001 and 39% vs. 24%, P = 0.004, respectively). Regarding the pulmonary function test results, only TLC < 85% of predicted was more frequent in the IM-TBI group than in the Co-TBI group (19% vs. 8%, P = 0.012) (Table 1).

### Characteristics of and risk factors for interstitial pneumonitis

During the follow-up period (median duration, 138.8 months), 44 patients developed IP, 19 cases of which were idiopathic (Tables 1 and 2). The median interval from the date of HSCT to the development of IP was 2.5 months (range, 0.2 to 34.4 months). Eighty percent of the patients with IP were diagnosed within 6 months after HSCT. The overall 1-year cumulative incidences of IP and idiopathic IP were 16.5% and 7.4%, respectively.

Regarding treatments, 41% (18/44) of patients in the total IP group and 53% (10/19) of those in the idiopathic IP group received corticosteroid. Concomitant infection was the main obstacle for the patients who were unable to receive corticosteroid. The IP-related respiratory failure rates were comparable between patients in the total IP cohort and idiopathic IP subgroup (55% [24/44] vs. 53% [10/19], respectively, P > 0.999). Among the survivors with follow-up lung function tests, 57% (4/7) had moderate to severe restrictive ventilator defect. The total IP-related mortality rate was 50% (22/44), which was similar to the idiopathic IP-related mortality rate (63% [12/19], P = 0.414).

**Figure 1:**
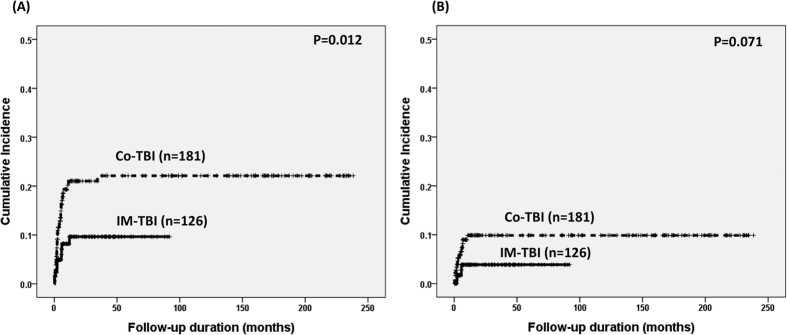
Cumulative incidence of (**A**) total interstitial pneumonitis and (**B**) idiopathic interstitial pneumonitis in terms of different TBI technique.

The risk factors for both total IP and idiopathic IP included non-CR at HSCT (19% vs. 12%, P = 0.020 and 9% vs. 5%, P = 0.050, respectively) and abnormal FVC before HSCT (24% vs. 12%, P = 0.010 and 13% vs. 5%, P = 0.012, respectively) (Tables 3 and 4). Although Co-TBI was a significant predictive factor for total IP (19% vs. 8%, P = 0.012, Table 3 and Fig. 1A), it only had borderline significance as a predictive factor for idiopathic IP (8% vs. 3%, P = 0.071, Table 4 and Fig. 1B).

In a multivariate Cox proportional hazards regression analysis for total IP, non-CR at HSCT, Co-TBI, and pre-HSCT abnormal FVC were independent predictors of higher incidence (Table 3); however, only Co-TBI was an independent prognostic factor for a higher incidence of idiopathic IP (Table 4).

**Figure 2:**
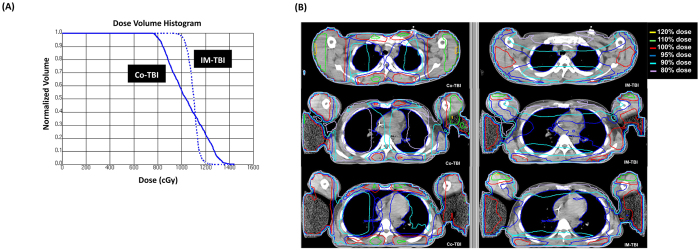
(**A**) The dose volume histogram showed the median dose was similar between these different techniques, but the lung dose distribution was much more homogenous for the IM-TBI plan. Solid line: Co-TBI; Dashed line: IM-TBI. (**B**) The iso-dose curve of lung revealed that there were more hot spots in the entrance and exit regions in the Co-TBI plan.

Comparisons of the lung doses for Co-TBI and IM-TBI are provided in Fig. 2A,B. The dose volume histogram showed that the lung dose was comparable between these two groups, but the lung dose distribution was much more homogenous for the IM-TBI plan than for the Co-TBI plan (Fig. 2A). The iso-dose curve also showed more hot spots in the entrance and exit regions in the Co-TBI plan (Fig. 2B).

### Prognostic impact in acute myeloid leukemia (AML) patients

In order to elucidate the prognostic impacts of different TBI techniques, we analyzed survival in the relative homogeneous group of patients with AML (n = 111). Univariate analyses of overall survival (OS) showed that age greater than 30 years (12.4 vs. 51.5 months, P = 0.037) and non-CR at HSCT (5.6 vs. 92.9 months, P < 0.001) were significant unfavorable prognostic factors. Other clinical parameters, including initial CNS involvement, unfavorable-risk cytogenetics, different TBI techniques, and GvHD (either acute or chronic), did not show any significant associations with survival in the univariate analyses. In a multivariate analysis of OS, only non-CR at HSCT was an unfavorable prognostic factor (relative risk [RR] 4.0, 95% confidence interval [CI] 2.4–6.7, P < 0.001, Table 5).

Regarding univariate analyses of relapse-free survival (RFS), age greater than 30 years (8.2 vs. 28.7, P = 0.034) and non-CR at HSCT (3.7 vs. 59.5 months, P < 0.001) were significant unfavorable prognostic factors. In resemblance with our multivariate analysis of OS, only non-CR at HSCT was an unfavorable prognostic factor in the multivariate analysis of RFS (RR 4.4, 95% CI 2.6–7.3, P < 0.001, Table 5).

## Discussion

Heterogeneous definitions of TBI-related IP have been used in the literature. In some studies, the definition of TBI-related IP included all causes of IP, including both infectious and idiopathic cases^19^, while other studies included idiopathic cases alone^16^. In addition, several confounding factors, such as underlying pulmonary disease with abnormal lung function, regimens of conditioning radiochemotherapy, GvHD, and concurrent infection, make it challenging to determine an accurate diagnosis for IP^17^. In this study, we analyzed both total IP and idiopathic IP to minimize the impact of the infectious causes.

The large majority of the patients who developed IP did so within 1 year after HSCT (97% for the IM-TBI group and 90% for the Co-TBI group, P = 0.407). Furthermore, 99% (65/66) of the survivors in the IM-TBI group and 97% of the survivors in the Co-TBI group (56/58, P = 0.599) were observed for more than 1 year, making it possible to detect most of the IP development after HSCT.

The possibility of developing IP is always a major concern for conditioning regimens that include TBI. Several patient-specific risk factors for IP were reported previously, including old age^15^,^20^, poor performance status^15^, and acute leukemia or myelodysplastic syndrome^15^,^16^. Each of these risk factors is non-modifiable. Post-HSCT events, such as GvHD, play important roles in IP development^15^,^16^,^17^,^18^,^20^,^21^,^22^,^23^, but they are closely associated with post-HSCT disease status and the primary care physicians’ treatment policies. The most important of the remaining modifiable risk factors for IP development are probably related to TBI itself^19^. The toxicity of TBI mainly depends on the total dose^18^, dose rates^15^,^19^, fractionation^18^, and application of lung shielding^17^,^18^. To the best of our knowledge, the present study included one of the largest patient cohorts. Furthermore, all patients received homogenous myeloablative conditioning regimens that consisted of TBI with a total dose of 12 Gy. In this study, we have provided the first report of a radiation dose-modulation method that was simple, cheap, effective, and easy to apply. The incidences of IP and idiopathic IP were lower in the IM-TBI group than in the Co-TBI group, even though patients in the IM-TBI group tended to have more risk factors for IP, including a higher prevalence of acute leukemia, more frequent abnormal pulmonary function test results before HSCT, and more frequent acute or chronic GvHD. In both univariate and multivariate analyses, IM-TBI was a favorable risk factor for IP development (Tables 3 and 4). Schneider and colleagues also commented that the reduced incidence of IP in patients receiving IM-TBI might be explained by the more homogenous lung dose distribution and the lower number of focal hot spots that were generated by the rice-bag compensators^23^,^25^.

The present investigation is one of the first studies to show that non-CR at HSCT is an unfavorable prognostic factor for IP, using both univariate and multivariate analyses. This might be explained by physicians’ attempts to induce more GvHD in patients without CR at HSCT, who were believed to have dismal outcomes. Previous studies concerning the association of TBI and IP were mainly based on mixed and various hematologic malignancies^1^,^12^,^15^,^16^,^17^,^18^,^19^,^20^,^21^,^22^,^24^,^25^,^26^,^27^,^28^. Thomas *et al*. were the first to describe TBI in transplantation for patients with AML, but their reports were limited to patients in first complete remission^2^,^3^. Although discussions about the side effects of TBI may have been included in other clinical trials that have compared TBI with various conditioning regimens, the patients in these trials may not have been representative of the general AML population^29^. In contrast, we enrolled consecutive patients—including those who were not in CR or who even suffered from relapse—so we could investigate the impact of CR on IP development.

Regarding the validity of our comparison of the two TBI techniques, a major concern is the different periods during which the techniques were performed. To eliminate the potential biases that might arise from these different periods, such as improvements in post-HSCT care, we additionally compared the IP rates of patients who received TBI-free conditioning chemotherapy composed of cyclophosphamide (60 mg · kg^−1^ · day^−1^ for 2 days) and busulfan (intravenous 3.2 mg · kg^−1^ · day^−1^ or oral 4 mg · kg^−1^ · day^−1^ for 4 days) (BuCy)^30^,^31^,^32^ during these two different eras. We divided the patients who were treated with BuCy during 1995–2014 into two groups, using March 1, 2007 as the splitting date. The 1-year cumulative incidence of IP in patients who received standard BuCy did not differ significantly between the two periods (before March 2007 vs. after March 2007, 8.6% vs. 12.2%, P = 0.420). These results suggest that the risk of IP was not reduced by changes in factors unrelated to IM-TBI, such as developments in post-HSCT care.

For both of the TBI techniques that were investigated in the present study, the incidences of IP and idiopathic IP were lower than has been recorded in some previous data^19^. These lower IP rates might be explained by the use of lung shielding, either with lung blocks (in Co-TBI) or with rice-bag compensators (in IM-TBI). Sampath *et al*. reported a reduction in the incidence of IP from 11 to 2.3% with lung shielding^33^. This result supports our finding that a reduced incidence of IP could be achieved by using shielding accessories to tailor the dose to the lung.

In the past 10 years, BuCy has increasingly been used as the first choice option. BuCy has been applied instead of a TBI-based conditioning regimen in consideration of the late toxicities of TBI and evidence that BuCy and TBI have comparable treatment outcomes^5^,^6^,^7^,^8^,^9^,^10^,^11^,^12^,^13^. However, TBI offers some irreplaceable benefits. For example, it does not spare sanctuary regions; it provides homogenous high doses, regardless of blood supply; it offers less cross-resistance with other antineoplastic agents; and it makes it possible to tailor the dose distribution by shielding or boosting sites of interest. In our practice, the TBI-containing conditioning regimen was often reserved for refractory disease, relapsed disease, or patients with CNS involvement (Table 1).

In univariate and multivariate analyses of the patients with AML, the use of different TBI techniques was not associated with any significant differences in OS or RFS. These findings indicate that the modified TBI technique, IM-TBI, was able to effectively reduce the risk of IP without compromising the treatment outcomes of patients with AML. However, because the study cohort included limited numbers of patients with acute lymphoblastic leukemia and lymphoma, survival analyses were not carried out in these two patient subgroups.

One of the limitations to this study was that we did not have complete performance status data. Weiner *et al*. stated that a pre-transplantation Karnofsky performance status score of less than 100% was a risk factor for IP in patients who received HSCT (RR 2.1, P < 0.0001)^15^. Unfortunately, as a consequence of the retrospective nature of this study, we were unable to analyze the associations between Karnofsky performance status score and IP development.

In conclusion, the rice-bag compensator-containing TBI technique (IM-TBI) may be capable of providing a substantially lower IP rate than the conventional lung-shielding TBI technique (Co-TBI). Additionally, in the subgroup of patients with AML, the IM-TBI technique may be able to provide OS and RFS rates that are similar to those associated with Co-TBI. Further efforts should be invested in developing new and modified techniques of tailoring dose distributions to sites of interest, with the ultimate goal of reducing the rates of late toxicities.

## Methods and Materials

### Patients

This study recruited a total of 307 consecutive patients who received HSCT with a TBI-based myeloablative conditioning regimen from 1995 to 2014 at the National Taiwan University Hospital, including both children and adults. All patients received a pre-HSCT baseline pulmonary function test, routine chest radiograph, and high resolution computed tomography (CT) of the lungs. The patients who received the two different techniques of TBI (IM-TBI and Co-TBI) were compared in terms of the following characteristics: age, definite diagnosis, pre-HSCT disease status, conditioning regimens, and pre-HSCT pulmonary function test results, including FEV1, FVC, TLC, and DLCO. This study was approved by the Research Ethics Committee of the National Taiwan University Hospital and was performed in accordance with the Declaration of Helsinki, including all relevant details. Every patient provided written informed consent.

### Conditioning radiochemotherapy and post-HSCT care

The majority of the patients (243/307, 79.1%) received conditioning radiochemotherapy that consisted of cyclophosphamide and TBI. Other conditioning regimens were composed of TBI and melphalan, fludarabine, etoposide, and/or cytarabine. The medications for GvHD prophylaxis included anti-thymocyte globulin, cyclosporine, methotrexate, and/or mycophenolate mofetil^34^. Trimethoprim/sulfamethoxazole was used for *pneumocystis jirovecii* pneumonia prophylaxis. After 2007, regular blood sampling tests for serum cytomegalovirus viral load were initiated after conditioning chemotherapy, and lasted for at least 6 months after HSCT. Pre-emptive anti-cytomegalovirus therapy was delivered if the serum cytomegalovirus viral load became elevated.

### Total body irradiation

Two techniques of TBI were used during different time periods in this study. After March 2007, the radiotherapy technique for TBI was switched from cobalt-60 teletherapy machines (Co-TBI) to linear accelerators with rice-bag compensators for intensity modulation (IM-TBI). Both techniques included two parallel opposed diagonal fields (40 × 40 cm), which were accomplished through rotation of the fully opened collimator by 45 degrees, to cover the entire body.

In the Co-TBI group, patients were treated with a cobalt-60 1.25 MeV teletherapy machine at a source-surface distance equaling 300 cm. Patients received 1.5 Gy per fraction for 3 to 4 days, to a total dose of 12 Gy, and were irradiated anteroposteriorly with a dose rate of 4.4–5.8 cGy/min. Adults were treated in a standing upright position, while children were treated in a reclining position^24^. To lower the exposure of the lungs, 1-cm Cerrobend blocks were placed on top of an acrylic box tray as a beam spoiler. The region of the chest wall under the shielding lung blocks would be boosted with electron beams of appropriate energy ranging from 6 to 9 MeV. The final lung dose would be reduced to 70 to 80% of the prescribed dose.

**Figure 3:**
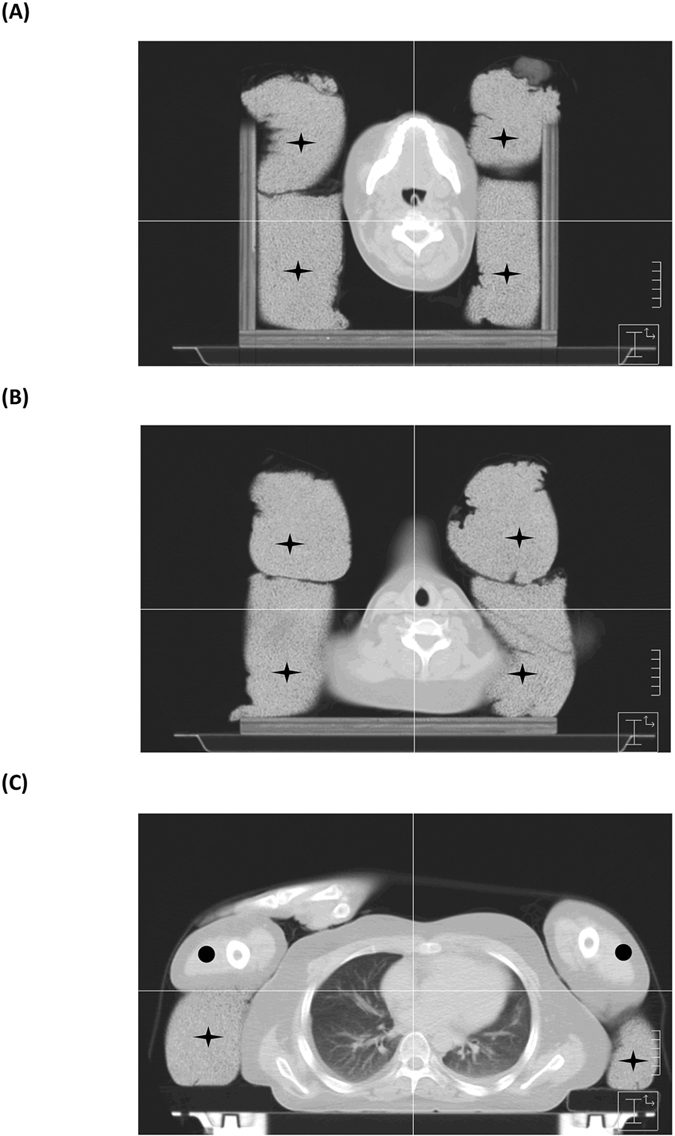
Axial views of simulation CT scan at (**A**) head, (**B**) neck, and (**C**) chest regions. Cross: rice-bags; Dot: arms.

In the IM-TBI group, TBIs were delivered with linear accelerators using 10 megavolt photons at source-axis distance equaling 380–470 cm. The total dose was 12 Gy, and was delivered in 8 fractions within 4 consecutive days. The dose rate was 5–10 cGy/min, depending on the patients’ body thickness. Patients lay in a supine position on a 32 cm-wide headboard with both arms positioned on the chest and the legs bent. We used the lateral diameter of the body at the level of the umbilicus as the reference thickness. If the reference thickness was more than 32 cm, additional bolus was applied on the bilateral outer sides of the headboard; on the other hand, polystyrene foam would be placed on the inner side of the headboard if the reference thickness was less than 32 cm. The rice-bag compensators were placed around the head, neck, shoulders, and chest to fill the interspace of these regions, and to decrease the variation of lateral body thickness along the patient axis (Fig. 3). The lung shielding was established using the rice bag and the patients’ arms as lateral compensators (Fig. 3C). A large spoiler screen made of 2-cm thick acrylic was placed within 10 cm of the patients’ surface to bring the surface dose to at least 90% of the prescribed dose. CT-based simulation and treatment planning were performed to ensure dose uniformity within ± 10%, as measured by thermoluminescent detectors that were placed at the bilateral eyes, neck, bilateral waist, umbilicus, lower back, and groin. The lungs were attenuated to a median dose of 10 Gy (range 9.6 to 10.8 Gy).

To compare the homogeneity of IM-TBI and Co-TBI, we simulated a three-dimensional plan for Co-TBI by inputting the Co-60 beam data into the Pinnacle Treatment Planning System, version 9.2.0.60009 (Philips Healthcare, USA). This allowed us to compare the dose volume histogram and iso-dose curve of the lung between the Co-TBI and IM-TBI groups.

### Interstitial pneumonitis

All patients with respiratory symptoms and abnormal chest radiograph findings received high resolution CT of the lungs. IP was defined as multilobar infiltrates on routine chest radiographs and CT in the absence of congestive heart failure, renal failure, or iatrogenic fluid overload^17^,^35^. The etiology of IP was investigated through a detailed examination of microbiological stains and cultures, as well as polymerase chain reaction tests for cytomegalovirus, *pneumocystis jiroveci* pneumonia, tuberculosis^36^,^37^, and other diseases. Around 25% of the patients received bronchoalveolar lavage or open lung biopsy^38^. If there was no clear pathogenic micro-organism, the IP was classified as idiopathic IP^16^.

### Cytogenetics

Bone marrow cells were harvested directly or after 1 to 3 days of unstimulated culture, as described previously^39^. Metaphase chromosomes were banded using the trypsin-Giemsa technique and karyotyped according to the International System for Human Cytogenetic Nomenclature. Cytogenetic findings were risk-stratified according to the modified Medical Research Council classification^40^.

### Statistical analysis

We compared the characteristics of the Co-TBI and IM-TBI groups using chi-square tests. OS was measured from the date of donor stem cell infusion (described as the date of HSCT elsewhere in this article) to the date of last follow-up or death from any cause, whereas relapse was defined as the reappearance of at least 5% leukemic blasts in bone marrow aspiration smears or new extramedullary leukemia in patients with a previously documented CR^41^. RFS was measured from the date of HSCT until relapse or death from any cause, whichever occurred first. OS, RFS, and cumulative incidence of IP were estimated using the Kaplan-Meier method. Multivariate Cox proportional hazards regression analyses were used to investigate independent prognostic factors for OS and RFS. The proportional hazards assumption (i.e., the constant hazards assumption) was examined by using time-dependent covariate Cox regression before conducting the multivariate Cox proportional hazards regression. A P-value < 0.05 was considered statistically significant. All statistical analyses were performed using SPSS software, version 20 (SPSS Inc., Chicago, IL, USA).

## Additional Information

**How to cite this article**: Chiang, Y. *et al*. Reduced incidence of interstitial pneumonitis after allogeneic hematopoietic stem cell transplantation using a modified technique of total body irradiation. *Sci. Rep*. **6**, 36730; doi: 10.1038/srep36730 (2016).

**Publisher’s note:** Springer Nature remains neutral with regard to jurisdictional claims in published maps and institutional affiliations.

## Figures and Tables

**Table 1:** Patient characteristics.

Characteristics	Co-TBI (%) (n = 181)	IM-TBI (%) (n = 126)	P-value
**Generation**			
Before March, 2007	181 (100)	0 (0)	
After March, 2007	0 (0)	126 (100)	
**Gender**			
Male	102 (56)	70 (56)	0.907
Female	79 (44)	56 (44)	
**Age (years)**			
<18	39 (22)	32 (25)	0.375
≥18	142 (78)	94 (75)	
**Underlying diagnosis**			
Acute leukemia	135 (75)	108 (86)	0.022
Others	46 (25)	18 (14)	
**Initial CNS involvement**			
Present	9 (5)	38 (30)	<0.001
Absent	172 (95)	88 (70)	
**Pre-transplantation response status**			
CR	116 (64)	79 (63)	0.811
Non-CR	65 (36)	47 (37)	
**Conditioning regimen**			
TBI and Cy	140 (77)	103 (82)	0.393
TBI and Others	41 (23)	23 (18)	
**Pre-transplantation pulmonary function test**			
FVC < 85% of predicted	28 (15)	26 (21)	0.286
FVC ≥ 85% of predicted	153 (85)	100 (79)	
FEV1 < 85% of predicted	36 (20)	32 (25)	0.266
FEV1 ≥ 85% of predicted	145 (80)	94 (75)	
TLC < 85% of predicted^a^	11 (8)	18 (19)	0.012
TLC ≥ 85% of predicted^a^	124 (92)	75 (81)	
DLCO < 70 of predicted^b^	23 (22)	19 (21)	0.999
DLCO ≥ 70 of predicted^b^	83 (78)	70 (79)	
**Acute GvHD**^c^			
No GvHD	113 (65)	43 (34)	<0.001
Grade I	19 (11)	21 (17)	
Grade II&III&IV	43 (24)	61 (49)	
**Chronic GvHD**^d^			
No GvHD	136 (76)	76 (61)	0.004
Limited/extensive GvHD	44 (24)	49 (39)	
**IP etiology**			
Infection	19	6	>0.999
Cytomegalovirus	9	1	0.411
*Pneumocystis jirovecii*	5	1	>0.999
Bacteria/atypical pathogen	2	3	0.069
Mixed	3	1	>0.999
Idiopathic (including possibly GvHD)	15	4	>0.999

Abbreviation: Co-TBI: total body irradiation with cobalt-60; IM-TBI: intensity-modulated total body irradiation; ALL: acute lymphoblastic leukemia; AML: acute myeloid leukemia; CNS: central nervous system; CR: complete remission; FVC: forced vital capacity; FEV1: forced expiratory volume in 1 second; TLC: total lung capacity; DLCO: diffusing capacity; GvHD: graft-versus-host disease; IP: interstitial pneumonitis.

^a^Available data of 93 patients in IM-TBI arm and 135 patients in Co-TBI arm.

^b^Available data of 89 patients in IM-TBI arm and 106 patients in Co-TBI arm.

^c^Available data of 125 patients in IM-TBI arm and 175 patients in Co-TBI arm.

^d^Available data of 125 patients in IM-TBI arm and 180 patients in Co-TBI arm.

**Table 2:** Characteristics of patients with interstitial pneumonitis.

UPN	Sex	Age	Diagnosis	CNS involvement	Conditioning	TBI technique	IP etiology	Time to IP (months)	Survival Status	OS (months)	Treatment†	IP related respiratory failure
1	Male	5	ALL	No	TBI + Cy	Co-TBI	CMV	1.2	Dead	1.6	A + V + M	Yes
2	Male	5	ALL	No	TBI + Cy	Co-TBI	Mixed	11.2	Dead	11.3	A + V + M	Yes
3	Male	5	NHL	No	TBI + Cy	Co-TBI	Idiopathic	2.3	Dead	2.8	A + M	Yes
4	Male	11	ALL	No	TBI + Cy	Co-TBI	Idiopathic	2.7	Dead	3.0	A + V + M	Yes
5	Male	15	ALL	No	TBI + Cy	Co-TBI	CMV	2.3	Dead	2.5	A + V + M	Yes
6	Male	16	AML	No	TBI + Cy	Co-TBI	Idiopathic	2.6	Dead	2.0	A + M	Yes
7	Female	16	AML	No	TBI + Cy	Co-TBI	CMV	1.1	Dead	4.2	A + V	No
8	Female	17	SAA	No	TBI + Cy	Co-TBI	Idiopathic	0.7	Alive	175.4	S	No
9	Female	18	AML	No	TBI + Bu + Cy	Co-TBI	Idiopathic	0.9	Dead	1.4	A + M	Yes
10	Female	18	AML	Yes	TBI + Cy	IM-TBI	PJP	2.3	Alive	88.4	A	No
11	Male	19	CML	Yes	TBI + Cy	IM-TBI	Bacteria/Atypical bacteria	6.0	Alive	67.4	A	No
12	Male	20	ALL	No	TBI + Cy	Co-TBI	Idiopathic	1.5	Dead	2.3	A + V + S + M	Yes
13	Male	20	AML	No	TBI + Cy	IM-TBI	PJP	1.1	Dead	1.4	A + S	Yes
14	Male	20	ALL	No	TBI + Cy + Etoposide	Co-TBI	Idiopathic	0.2	Dead	0.9	A + M	Yes
15	Male	21	ALL	Yes	TBI + Cy	IM-TBI	CMV	0.2	Dead	34.7	A + V + S + M	Yes
16	Male	21	AML	No	TBI + Cy	Co-TBI	Idiopathic	6.8	Alive	191.4	A + S	No
17	Male	21	ALL	No	TBI + Cy	Co-TBI	CMV	2.0	Dead	3.1	A + V + S + M	Yes
18	Female	22	AML	No	TBI + Cy	Co-TBI	Idiopathic	6.1	Alive	155.4	A	No
19	Male	22	CML	No	TBI + Cy	Co-TBI	Idiopathic	2.0	Alive	236.7	A + V	No
20	Male	23	ALL	No	TBI + Cy + Fludarabine	Co-TBI	PJP	5.4	Dead	61.0	A + S	No
21	Male	24	AML	No	TBI + Cy	Co-TBI	Idiopathic	10.3	Dead	15.5	A + V + S	No
22	Male	25	AML	No	TBI + Cy	Co-TBI	CMV	5.2	Dead	5.9	A + V	Yes
23	Female	25	ALL	No	TBI + Cy	Co-TBI	Idiopathic	0.8	Dead	64.2	A	No
24	Female	27	ALL	No	TBI + Cy	IM-TBI	Bacteria/Atypical bacteria	0.2	Dead	3.6	A	No
25	Male	28	ALL	Yes	TBI + Cy	IM-TBI	Idiopathic	5.8	Alive	87.4	A + V + S	No
26	Male	28	AML	No	TBI + Cy	Co-TBI	PJP	7.6	Alive	124.0	A	No
27	Male	29	AML	No	TBI + Cy	Co-TBI	PJP	34.4	Dead	34.8	A + S	Yes
28	Female	29	MM	No	TBI + Melphalan	Co-TBI	Mixed	2.6	Dead	9.3	A + V	No
29	Male	32	CML	No	TBI + Cy	Co-TBI	CMV	2.3	Dead	2.4	A + V + M	Yes
30	Male	34	MM	No	TBI + Cy + Etoposide	Co-TBI	CMV	1.6	Dead	2.0	A + V	Yes
31	Female	37	CML	No	TBI + Cy + Etoposide	Co-TBI	PJP	2.9	Dead	3.0	A + V	Yes
32	Male	38	NHL	No	TBI + Cy	IM-TBI	Idiopathic	5.7	Alive	20.0	A + S	No
33	Female	39	AML	No	TBI + Cy + Clofarabine	IM-TBI	Idiopathic	2.3	Dead	3.5	A + S	Yes
34	Male	39	AML	No	TBI + Cy + Etoposide	Co-TBI	Idiopathic	6.5	Dead	8.2	A + S + V + M	Yes
35	Male	39	AML	No	TBI + Cy	Co-TBI	CMV	0.7	Dead	59.4	A + V	Yes
36	Male	40	ALL	No	TBI + Cy	Co-TBI	CMV	4.2	Dead	5.2	A + V	No
37	Female	41	ALL	No	TBI + Cy	Co-TBI	Bacteria/Atypical bacteria	2.1	Alive	199.4	A + V	No
38	Female	41	CML	No	TBI + Cy	Co-TBI	Idiopathic	3.9	Dead	5.3	A + S	Yes
39	Male	41	CML	No	TBI + Cy	Co-TBI	Bacteria/Atypical bacteria	1.7	Alive	207.8	A	No
40	Female	42	AML	No	TBI + Cy	Co-TBI	PJP	5.5	Dead	5.7	A + S	Yes
41	Female	46	AML	Yes	TBI + Cy + Etoposide	Co-TBI	PJP	4.8	Dead	5.2	A + S	Yes
42	Male	48	AML	No	TBI + Cy	Co-TBI	Idiopathic	5.1	Dead	5.6	A + V	Yes
43	Male	54	ALL	No	TBI + Cy	IM-TBI	Bacteria/Atypical bacteria	12.0	Alive	32.3	A + V + S	No
44	Female	56	ALL	No	TBI + Cy	IM-TBI	Idiopathic	2.1	Dead	4.9	A + S	No

Abbreviation: UPN: unique patient number; ALL: acute lymphoblastic leukemia; AML: acute myeloid leukemia; SAA: severe aplastic anemia; MM: multiple myeloma; NHL: non-Hodgkin lymphoma; CML: chronic myeloid leukemia; CNS: central nervous system; TBI: Total body irradiation; Bu: Busulfan; Cy: Cyclophosphamide; IP: interstitial pneumonitis; CMV: cytomegalovirus; PJP: *pneumocystis jirovecii* pneumonia; Co-TBI: total body irradiation with cobalt-60; IM-TBI: intensity-modulated total body irradiation; OS: overall survival.

^†^A: Antimicrobials, including antibacterials and antifungals; V: antivirals, including ganciclovir, foscarnet, and human cytomegalovirus immunoglobulin; S: corticosteroid; M: mechanical ventilation.

**Table 3:** Risk factors of total interstitial pneumonitis.

Characteristic	Univariate		Multivariate			
	Total IP (%)	P-value	RR	95% CI		P-value
				Lower	Upper	
**Gender**						
Male vs. Female	29/172 (17) vs. 15/135 (11)	0.169				
**Age (years)**						
<18 vs. ≥ 18	10/89 (12) vs. 34/218 (15)	0.405				
**Underlying diagnosis**						
Acute leukemia vs. Others	33/243 (14) vs. 11/64 (17)	0.620				
**Initial CNS involvement**						
Yes vs. No	5/47 (11) vs. 39/260 (15)	0.404				
**Pre-transplantation response status**						
Non-CR vs. CR	21/112 (19) vs. 23/195 (12)	0.020	2.1	1.2	3.9	0.015
**Conditioning chemotherapy**						
Cyclophosphamide alone vs. Others	35/243 (15) vs. 9/64 (14)	0.802				
**RT technique**						
Co-TBI vs. IM-TBI	34/181 (19) vs. 10/126 (8)	0.012	2.7	1.3	5.6	0.007
**Pre-transplantation pulmonary function test**						
FVC < 85% vs. ≥ 85% of predicted	13/54 (24) vs. 31/253 (12)	0.010	2.6	1.0	6.3	0.038
FEV1 < 85% vs. ≥ 85% of predicted	13/68 (19) vs. 31/239 (13)	0.101	1.1	0.5	2.8	0.780
**Acute GvHD**						
Grade 0/I vs. Grade II/III/IV	27/196 (14) vs. 15/104 (14)	0.881	0.7	0.4	1.4	0.360
**Chronic GvHD**						
Without vs. With	30/212 (14) vs. 14/93 (15)	0.634	1.1	0.6	2.3	0.715

Abbreviation: IP: interstitial pneumonitis; Co-TBI: total body irradiation with cobalt-60; IM-TBI: intensity-modulated total body irradiation; CNS: central nervous system; CR: complete remission; FVC: forced vital capacity; FEV1: forced expiratory volume in 1 second; TLC: total lung capacity; DLCO: diffusing capacity; GvHD: graft-versus-host disease; RR, relative risk; CI: confidence interval.

**Table 4:** Risk factors of idiopathic interstitial pneumonitis.

Characteristic	Univariate		Multivariate			
	Idiopathic IP (%)	P-value	RR	95% CI		P-value
				Lower	Upper	
**Gender**						
Male vs. Female	12/172 (7) vs. 7/135 (5)	0.524				
**Age (years)**						
<18 vs. ≥ 18	5/89 (6) vs. 14/218 (6)	0.859				
**Underlying diagnosis**						
Acute leukemia vs. Others	14/243 (6) vs. 5/64 (7)	0.623				
**Initial CNS involvement**						
Yes vs. No	1/47 (2) vs. 18/260 (7)	0.194				
**Pre-transplantation response status**						
Non-CR vs. CR	10/112 (9) vs. 9/195 (5)	0.050	2.4	0.9	6.1	0.066
**Conditioning chemotherapy**						
Cyclophosphamide alone vs. Others	15/243 (6) vs. 4/64 (6)	0.810				
**RT technique**						
Co-TBI vs. IM-TBI	15/181 (8) vs. 4/126 (3)	0.071	3.2	1.0	10	0.042
**Pre-transplantation pulmonary function test**						
FVC < 85% vs. ≥ 85% of predicted	7/54 (13) vs. 12/253 (5)	0.012	3.0	0.8	12	0.109
FEV1 < 85% vs. ≥ 85% of predicted	7/68 (10) vs. 12/239 (5)	0.069	1.4	0.3	5.4	0.661
**Acute GvHD**						
Grade 0/I vs. Grade II/III/IV	11/196 (6) vs. 7/104 (7)	0.688	0.7	0.3	1.8	0.433
**Chronic GvHD**						
Without vs. With	12/212 (6) vs. 7/93 (8)	0.916	0.9	0.7	2.5	0.855

Abbreviation: IP: interstitial pneumonitis; Co-TBI: total body irradiation with cobalt-60; IM-TBI: intensity-modulated total body irradiation; CNS: central nervous system; CR: complete remission; FVC: forced vital capacity; FEV1: forced expiratory volume in 1 second; TLC: total lung capacity; DLCO: diffusing capacity; GvHD: graft-versus-host disease; RR, relative risk; CI: confidence interval.

**Table 5:** Survival analysis of the OS and RFS in AML patients.

	OS						RFS					
	Univariate		Multivariate				Univariate		Multivariate			
	Median (months)	P-value	RR	95% CI		P-value	Median (months)	P-value	RR	95% CI		P-value
				Lower	Upper					Lower	Upper	
Age†	12.4 vs. 51.5	0.037	1.317	0.803	2.160	0.275	8.2 vs. 28.7	0.034	1.265	0.770	2.077	0.353
Initial CNS involvement§	93.0 vs. 16.8	0.297					59.5 vs. 13.1	0.267				
KaryotypeΨ	15.9 vs. 18.8	0.407					14.8 vs.13.5	0.309				
Pre-HSCT statusζ	5.6 vs. 92.9	<0.001	4.032	2.415	6.711	<0.001	3.7 vs. 59.5	<0.001	4.367	2.618	7.299	<0.001
RT technique*	15.9 vs. 18.8	0.956	1.342	0.787	2.290	0.280	13.1 vs. 15.4	0.809	1.454	0.854	2.475	0.168
Acute GvHD%	9.6 vs. 20.4	0.318					6.9 vs. 18.4	0.422				
Chronic GvHD@	24.6 vs. 16.8	0.858					15.5 vs. 13.5	0.924				

Abbreviation: OS, overall survival; RFS, relapse-free survival; RR, relative risk; CI: confidence interval.

^†^Age ≧ 30 vs. Age < 30 (reference).

^§^Initial with CNS involvement vs. not (reference).

^Ψ^Unfavorable cytogenetics vs. others (reference).

^ζ^Non-CR vs. CR (reference).

^*^IM-TBI vs. Co-TBI (reference).

^%^Grade II/III/IV vs. Grade 0/I (reference).

^@^With cGvHD vs. without (reference).
